# Importance of Classical Morphology in the Diagnosis of Myelodysplastic Syndrome

**DOI:** 10.4084/MJHID.2015.035

**Published:** 2015-05-01

**Authors:** Rosangela Invernizzi, Federica Quaglia, Matteo Giovanni Della Porta

**Affiliations:** Department of Internal Medicine, University of Pavia, IRCCS Policlinico San Matteo Foundation, Pavia, Italy

## Abstract

Myelodysplastic syndromes (MDS) are hematopoietic stem cell disorders characterized by dysplastic, ineffective, clonal and neoplastic hematopoiesis. MDS represent a complex hematological problem: differences in disease presentation, progression and outcome have necessitated the use of classification systems to improve diagnosis, prognostication, and treatment selection. However, since a single biological or genetic reliable diagnostic marker has not yet been discovered for MDS, quantitative and qualitative dysplastic morphological alterations of bone marrow precursors and peripheral blood cells are still fundamental for diagnostic classification. In this paper, World Health Organization (WHO) classification refinements and current minimal diagnostic criteria proposed by expert panels are highlighted, and related problematic issues are discussed. The recommendations should facilitate diagnostic and prognostic evaluations in MDS and selection of patients for new effective targeted therapies. Although, in the future, morphology should be supplemented with new molecular techniques, the morphological approach, at least for the moment, is still the cornerstone for the diagnosis and classification of these disorders.

## Introduction

Myelodysplastic syndromes (MDS) are clonal hematopoietic stem cell disorders characterized by dysplastic, ineffective and neoplastic hematopoiesis. The risk of evolution to acute myeloid leukemia (AML) is variable, and the clinical outcome is greatly heterogeneous. Therefore, MDS constitute a complex hematological problem that gives rise to difficulties in diagnosis and therapeutic decision-making.^1^ Since a single biological or genetic reliable diagnostic marker has not yet been discovered for MDS, quantitative and qualitative dysplastic alterations of bone marrow precursors and of peripheral blood cells are still fundamental for diagnostic classifications.^2^ While the detection of increased blast cells may facilitate the diagnosis in advanced forms, in the early forms, especially with modest morphological abnormalities, a correct diagnosis is based mainly on the exclusion of other diseases. Some bone marrow failure syndromes can indeed mimic the MDS,^3^,^4^ and the formulation of a correct diagnosis is fundamental for both prognostic evaluation and therapeutic approach.

In this review the meaning of morphology in MDS is examined; World Health Organization (WHO) classification refinements and current minimal morphological criteria for defining dysplastic involvement are highlighted, and several problematic issues are discussed.

## Diagnosis and Classification

Currently, the reference classification of MDS is still the WHO classification, published in 2001 and updated in 2008.^5^–^7^ This classification system is based on an integrated multidisciplinary approach that uses all available information (morphology, cytochemistry, immunophenotype, genetics, clinical aspects) to define biologically homogeneous and clinically relevant entities, that can be usefully applied in clinical practice. The WHO classification improved the prognostic value of the former FAB classification,^8^ by recognizing more specific categories on the basis of cytogenetic findings as well as cellular morphology and allowed to evaluate more accurately emerging therapies that target specific genetic abnormalities.^9^,^10^

The suspicion of MDS arises on the basis of an abnormal blood count with evidence of different combinations of anemia, neutropenia, and thrombocytopenia in an appropriate clinical setting. Anemia is often macrocytic, associated with a significantly reduced reticulocyte count. Obviously, all causes of reactive cytopenia/dysplasia should be excluded as well as other clonal stem cell disorders and congenital abnormalities (Table 1). The minimal diagnostic criteria for MDS include the presence of bone marrow specific alterations, i.e. one or more of the following characteristics: dysplasia in at least 10% of at least one of the major hematopoietic lineages, at least 15% ring sideroblasts or 5–19% myeloblasts in bone marrow smears. Certain chromosomal abnormalities detected by conventional karyotyping or FISH in the presence of a refractory cytopenia, but no morphological evidence of dysplasia, are considered presumptive evidence for MDS (Table 2).^6^,^11^,^12^ Since morphology alone is often insufficient to reach a final diagnosis, it should be integrated, but not replaced, by other investigations such as flow cytometry, molecular studies, in vitro culture of hematopoietic progenitors.^2^,^13^,^14^ However, if multilineage dysplasia, chromosomal aberrations and proof of clonality are absent, the diagnosis may be difficult.

On the basis of the proportion of peripheral blood and bone marrow blasts, defined by a morphological examination, two broad categories of MDS are recognized: forms with <2% peripheral blood blasts and <5% bone marrow blasts (lower risk subtypes), including refractory cytopenias with unilineage dysplasia (RCUD), refractory anemia with ring sideroblasts (RARS), refractory cytopenia with multilineage dysplasia (RCMD), myelodysplastic syndrome-unclassified (MDS-U) and MDS associated with isolated del(5q), and forms characterized by at least 2% peripheral blood blasts and/or at least 5% bone marrow blasts (higher risk subtypes), including refractory anemia with excess blasts-1 (RAEB-1) and RAEB-2 (Table 3). Chronic myelomonocytic leukemia (CMML), characterized by persistent monocytosis, is placed into the category of myelodysplastic/myeloproliferative neoplasms together with atypical chronic myeloid leukemia (ACML), *BCR-ABL1* negative, juvenile myelomonocytic leukemia (JMML) and refractory anemia with ring sideroblasts associated with marked thrombocytosis (RARS-T), which is still a provisional entity.^15^,^16^

## Morphological Features

The diagnosis of MDS is mainly based on morphological findings of peripheral blood and bone marrow.^17^–^20^ Morphological examination has several advantages: it is a simple, technically easy, not expensive method, which gives quick results; moreover, it has prognostic importance, and should be supplemented, but not replaced, by other tests. The morphological examination requires peripheral blood smear, bone marrow aspirate, and bone marrow trephine biopsy.

Peripheral blood and bone marrow specimens should be collected before any definitive therapy. No case of MDS should be reclassified while the patient is on growth factor therapy. Since prolonged exposure to anticoagulants can cause artifacts, the slides for the assessment of dysplasia should be made from freshly obtained specimens. On bone marrow aspirate smears and/or biopsy touch preparations, MGG or similar staining and iron staining could possibly, but not necessarily, be supplemented by cytochemical dyes to identify bone marrow cells and maturation stages: myeloperoxidase and Sudan black detect myeloid cells by staining cytoplasmic granular contents and better identify Auer rods, periodic acid-Schiff detects lymphocytic cells and certain abnormal erythroid cells by staining cytoplasmic glycogen, esterases distinguish myelocytic from monocytic maturation stages. On bone marrow aspirates, the cellularity should be enough to perform a 500 cells differential count, whereas, on peripheral blood smears, a differential count of 200-cell leukocyte is recommended. The blood and marrow smears should be examined for the percentages of blasts, dysplastic cells and ring sideroblasts. At least 100 erythroblasts, 100 granulocytic cells, and 30 megakaryocytes should be evaluated.^6^

## Assessment of Blasts

An increase of blast cells has to be considered as a sign of myelodysplasia. An International Working Group on Morphology of MDS (IWGM-MDS) of hematopathologists and hematologists, in order to improve diagnostic accuracy, agreed on some recommendations for the definition and enumeration of blasts.^21^ First, blast percentage should be determined by visual inspection. Flow cytometric assessment of CD34+ cells is not recommended, as not all blasts express CD34 antigen and flow cytometry analysis can be affected by peripheral blood dilution of the sample.^6^ Myeloblasts, monoblasts, promonocytes, and megakaryoblasts should be counted as blasts; dysplastic megakaryocytes and proerytrhoblasts must not be counted as blasts except in the rare cases of “pure” acute erythroleukemia. Blast lineage could be assessed by flow cytometry, cytochemistry or immunocytochemistry. In severely cytopenic patients, buffy coat smears of peripheral blood may facilitate performing the differential count. The diagnostic and prognostic importance of an accurate count of the blasts should be emphasized.^22^ According to WHO, 20% bone marrow or peripheral blood blasts is the threshold for the diagnosis of AML, whereas, according to the revised International Prognostic Scoring System, the forms with <2% bone marrow blasts are to be distinguished from those with >2% blasts, as they have a better prognosis.^23^ Moreover, they were included in the MDS-U subtype patients with 1% blasts in the blood and fewer than 5% blasts in the bone marrow.^24^

**Figure 1:**
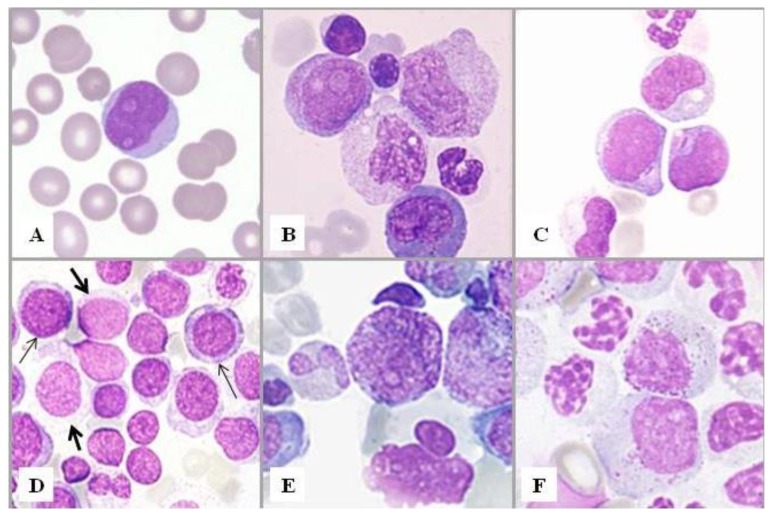
Bone marrow smears. Blast cells and dysplastic promyelocytes. **A)** A blast with agranular cytoplasm. **B)** A blast with some azurophilic granules scattered in its cytoplasm. This type of blasts is classified as granular irrespective of the number of granules. A granular blast can be distinguished from a promyelocyte by the less degree of chromatin clumping and the lack of a clear paranuclear area. Also apparent are, from top to bottom, a lymphocyte, a late erythroblast, two myelocytes, an agranular neutrophil with band nucleus and an eosinophil. **C)** Two blasts with a single Auer body in their cytoplasm. In MDS, the presence of an Auer body in a blast allows the automatic diagnosis of RAEB-2, according to WHO criteria. **D)** Agranular blasts (thick arrows) can be distinguished from early erythroid precursors (thin arrows) by the less degree of chromatin clumping and the smaller size. **E)** A hypergranular promyelocyte. **F)** Promyelocytes with scanty primary granules. Note also late granulocytic cells showing abnormal chromatin clumping and decreased secondary granules.

Blasts have variable size, ovoid or irregularly outlined nuclei with loose chromatin pattern and variable number of nucleoli, basophilic cytoplasm, with the absence of an evident Golgi zone. They are defined as granular or agranular and may contain Auer rods, whose presence allows the automatic diagnosis of RAEB-2. Myeloblasts showing strongly basophilic cytoplasm could be misinterpreted as immature erythroid precursors. Erythroid precursors, however, have relatively mature clumped chromatin and are often larger than myeloblasts at early stages. Granular blasts should be distinguished from normal or dysplastic promyelocytes. Promyelocytes are usually characterized by a well recognizable Golgi zone; dysplastic promyelocytes, however, are often hyper- or hypogranulated and may present a less evident Golgi area than normal promyelocytes (Figure 1).

It is worth noting that in the forms with recurrent cytogenetic abnormalities, such as t(8;21)(q22;q22), inv(16)(p13.1q22) or t(16;16)(p13.1;q22) and t(15;17)(q22;q12) the diagnosis of AML should be made even with fewer than 20% bone marrow blasts. These forms are considered clinical-pathological-genetic entities with peculiar features.

## Assessment of Monocytic Cells

The IWGM-MDS also defined the different maturation stages of monocytic cells.^25^ A promonocyte differs from a monoblast for the irregular nuclear outline but has similar immature chromatin pattern; it is a blast equivalent and should be counted as such. Thus, the distinction between a monoblast and a promonocyte has no practical importance as they are regarded as having the same significance. An atypical/immature monocyte is characterized by a more condensed chromatin pattern and less evident nucleoli, but its distinction from a promonocyte can be very difficult. Monocytic cells can be better identified with the nonspecific esterase reaction. Monoblasts and promonocytes, however, are rare in MDS, and their presence is rather indicative of CMML or AML with monocytic differentiation.

## Assessment of Dysplasia

The precise recognition and quantification of dysplasia is critical for a correct application of the WHO classification for the following main reasons: WHO proposal introduced uni- versus multilineage dysplasia as a diagnostic criterion in MDS with fewer than 5% bone marrow blasts, increasing the prognostic value of the classification;^26^,^27^ the finding, in an appropriate clinical setting, of dysplastic morphological alterations in at least 10% of the cells of at least one myeloid lineage is the most important criterion for the diagnosis of RCUD. This subtype is rather difficult to recognize because of the minimal percentage of blasts in the bone marrow and the low incidence of chromosome abnormalities.^28^,^29^

**Figure 2:**
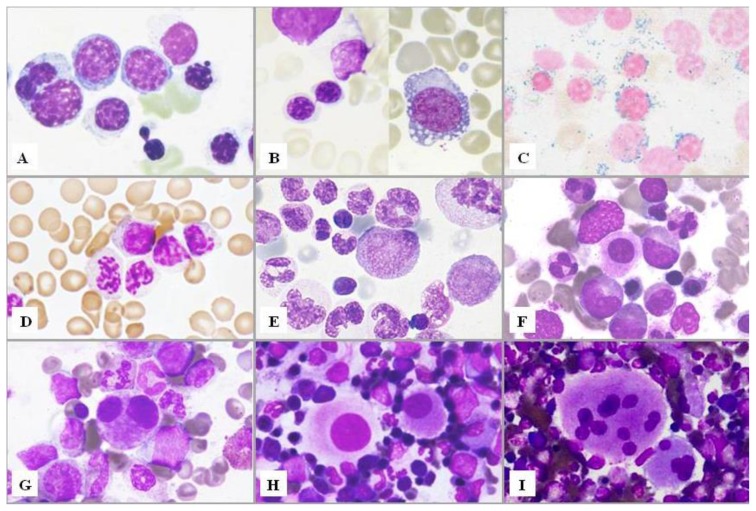
Bone marrow smears. Myelodysplastic features in hematopoietic cell lineages. **A)** Dyserythropoiesis. Erythroid hyperplasia with marked morphological abnormalities: megaloblastoid features; a trinucleated erythroblast (left); an erythroblast containing a Howell-Jolly body and an erythroblast with curiously lobulated nucleus. Late erythroblasts show ill-defined borders. **B)** Dyserythropoiesis. Left, internuclear bridge; right, a proerythroblast with vacuolated cytoplasm. **C)** Dyserythropoiesis. Perls’ staining shows ring sideroblasts with numerous positive granules surrounding a third or more of the circumference of the nucleus. **D)** Dysgranulopoiesis. Neutrophils with nuclear hypolobation (acquired Pelger-Hüet anomaly), abnormal chromatin clumping and agranular cytoplasm. **E)** Dysgranulopoiesis. Anisocytosis of neutrophils that show giant nuclear segments of bizarre shape. Centre, note a promyelocyte with pink inclusions in its cytoplasm. **F)** Dysmegakaryopoiesis. A micromegakaryocyte of about the size of the surrounding myeloid cells with scanty granular cytoplasm. **G)** Dysmegakaryopoiesis. A small binucleate megakaryocyte. **H)** Dysmegakaryopoiesis. Megakaryocytes with a single large round or oval nucleus and granular cytoplasm. **I)** Dysmegakaryopoiesis. Megakaryocytes with many round separate nuclei.

The dysplastic abnormalities of the cell nucleus and/or cytoplasm to be taken into account are listed in Table 4 and illustrated in Figure 2. Whereas variable degrees of dyserythropoiesis are commonly observed in various hematological, as well as non-hematological disorders, the morphological abnormalities of the granulocytic and megakaryocytic series are more specific and significant for the diagnosis. However, no single morphological finding is diagnostic for MDS, that sometimes remains a diagnosis of exclusion.

## Dysgranulopoiesis

Hypo-agranularity of neutrophils is considered a highly specific dysplastic feature; usually, it is more evident in peripheral blood smears and better assessable with Sudan black or peroxidase reaction.^30^ According to the recently published IWGM-MDS proposal for refining the definition of dysgranulopoiesis, neutrophils could be recognized as dysplastic in the presence of one of the following morphological features: at least 2/3 reduction of the content of granules, pseudo Pelger anomaly of the nucleus, not-Pelger abnormalities of nuclear segmentation, macropolycytes, abnormal clumping of the chromatin and the presence of more than four nuclear projections.^31^

## Dysmegakaryopoiesis

Micromegakaryocytes are highly specific for dysmegakaryopoiesis, but there is still no consensus on their definition. It is recommended to consider as micromegakaryocyte a megakaryocyte of about the size of the surrounding myeloid cells, with scanty granular cytoplasm. Other categories of dysplastic megakaryocytes are illustrated in Figure 2: medium sized megakaryocytes with a single, ovoid, eccentric nucleus, pathognomonic of the 5q- syndrome; or with 2 nuclei of similar or different size, close one to another; mature megakaryocytes with numerous small round separated nuclei.

## Dyserythropoiesis and Ring Sideroblasts

As already mentioned, morphological abnormalities of erythroid cells, as megaloblastic features and non-round nuclei, are commonly observed in many hematological as well as non-hematological disorders, and have a low diagnostic power. Only ring sideroblasts are considered highly specific dysplastic changes. Recommendations for the definition of ring sideroblasts have been provided by the IWGM-MDS.^21^ They are defined as erythroblasts characterized by at least 5 siderotic granules surrounding at least a third of the nuclear circumference, as a result of the iron accumulation within mitochondria, including some deposited as mitochondrial ferritin.^32^ A high microscopic magnification is necessary to distinguish these granules. In some cases, ring sideroblasts constitute <15% of erythroid precursors: in such cases the diagnosis of MDS with RS would not be possible. However, ring sideroblasts would be considered as unequivocal expression of dyserythropoiesis. On the contrary, type 1 sideroblasts, characterized by <5 siderotic granules, are also present in the normal bone marrow, whereas type 2 sideroblasts show at least five non-perinuclear siderotic granules. In type 1 and type 2 sideroblasts, siderotic granules represent aggregates of ferritin molecules that are stored in lysosomes.

## Erythroid Predominant MDS (MDS-E)

Recently, the term of MDS-E or MDS Ery has been proposed to indicate forms of MDS with marked erythroid hyperplasia. Marked erythroid hyperplasia (50% or greater) with or without left-shifted erythroid maturation can be seen in approximately 15% of patients with MDS and is often associated with the presence of ring sideroblasts.^33^ In this condition, the count of blasts should be performed on non-erythroid cells, excluding lymphocytes and plasma cells, and for the diagnosis of MDS, it should be lower than 20%. There is an ongoing discussion regarding the subclassification of MDS-E since low-risk MDS such as RA may be upgraded to a higher risk category if blasts were calculated as a percentage of non-erythroid cells.^34^,^35^ Thus, once the diagnosis of MDS is established, blast enumeration should be derived from all nucleated marrow cells. On the other hand, similar demographic and laboratory characteristics were reported in MDS-E in comparison with MDS cases with less than 50% erythroid precursors.

## Problematic Issues

The problems in the morphological diagnosis of MDS are mainly due to the non-specificity of dysplastic changes. Morphological alterations may be observed even in healthy bone marrow and in patients with non-clonal disorders; moreover, poor quality of marrow specimens and various artifacts may cause misinterpretation. On the other hand, recent studies have demonstrated discrepancy in morphological diagnosis in rather high proportions of cases as well as low reproducibility of the WHO 2008 criteria. Unfortunately, unanimous agreement on the type of morphological alterations that characterize MDS and on the threshold to be considered is still missing.^36^

Several studies have addressed the impact of the single morphological abnormalities and the degree of dysplasia on prognosis, and grading systems have been proposed to increase the diagnostic accuracy of MDS.^26^,^29^,^37^–^39^

A Japanese- German study concerning patients with MDS without excess blasts, 5q-syndrome excluded, showed the adverse prognostic significance of three parameters: the presence of at least 10% of micromegakaryocytes, dysmegakaryocytopoiesis > 40% and dysgranulopoiesis >10%. The authors suggested using these threshold values for the identification of multilineage dysplasia^26^. In a very detailed cytomorphological study on 3156 patients of the Düsseldorf register, no differences were observed in the frequency of dysplastic changes in relation to the WHO subtype of MDS and no single morphological abnormality had prognostic significance. Also, these authors recommended using 40% as a threshold value for dysmegakaryopoiesis.^40^

On the other hand, dysplastic features may also be observed in the normal bone marrow, as reported by some authors in the late ‘90s.^41^,^42^ A more recent work has shown dysgranulopoiesis >10% in 46% of the bone marrow aspirates from 120 healthy donors, with multilineage dysplasia in 26% of the subjects; however, the counting of cells with pseudo Pelger anomaly and micromegakaryocytes did not exceed 10% and total dysmegakaryopoiesis 40%. The concordance rate between the four investigators was modest in dysgranulopoiesis but poor in dyserythropoiesis and dysmegakaryopoiesis; raising the threshold from 10% to an arbitrary 20% for all lineages led to a higher concordance rate. In conclusion, the 10% cut-off for dyshematopoietic cells is questionable in patients without cytopenia and should be revised for future consensus recommendations.^43^ Interestingly, another study showed discordance in the morphological diagnosis between the reference and peripheral centers in 12% of 915 MDS cases referred to MD Anderson Cancer Center, with a majority reclassified as having higher-risk disease with implications for therapy selection and prognosis calculation.^44^ Finally, a Spanish group showed a poor reproducibility of the WHO criteria for cases with 5–9% marrow blasts or up to 1% circulating blasts as well as for the percentage of dysplastic erythroid cells.^45^

It should be emphasized the possible role of the barriers that can hinder a correct diagnostic definition: poor quality of marrow specimen, lack of clinical information, lack of available cytogenetic results, inter-observer variability in the assessment of dysplasia.^46^ The application of well codified reproducible criteria could allow a more objective morphological evaluation, and thus a correct implementation of the WHO classification.

## Morphological Score

In a retrospective study of 318 patients with MDS, a group of patients with other types of non-clonal cytopenias used as pathological controls, and a group of normal subjects, bone marrow hematopoietic cells were carefully examined and classified according to their nuclear and cytoplasmic morphological alterations to identify minimal reproducible morphological criteria to define marrow dysplasia and to evaluate the prognostic relevance of the degree of dysplasia.^47^ The most discriminant morphological features for dyserythropoiesis, dysgranulopoiesis and dysmegakaryopoiesis were identified. For each parameter, the optimal cut-off value to discriminate between MDS and controls and the weight in the recognition of BM dysplasia were determined to develop a score for defining minimal morphological criteria for MDS (Table 5). This score showed high sensitivity and specificity (>90%). The diagnostic value and reproducibility of the proposed criteria were independently validated (Table 6). There was a high inter-operator agreement, especially for patients with excess blasts. Very interestingly, erythroid score value did not significantly affect survival while granulocytic or megakaryocytic score levels had a significant effect on overall survival. Also, multilineage dysplasia showed an independent unfavorable prognostic value. Moreover, a close association was found between ring sideroblasts and *SF3B1* mutations and between severe granulocytic dysplasia and mutations of *ASXL1*, *RUNX1*, *TP53* and *SRSF2* genes.

In conclusion, this morphological score improving the objectivity and reproducibility of microscopic analysis might be very useful in the work-up of patients with suspected MDS. On the other hand, prognostic systems including the evaluation of the degree of bone marrow dysplasia should be adopted for clinical decision-making.

## Histopathology

A bone marrow trephine biopsy may increase the diagnostic accuracy and help in refining the prognostic scoring system for MDS. It provides information on cellularity and stroma and is essential for the identification of MDS with fibrosis and hypoplastic MDS.^48^–^52^ In these peculiar entities (10–15% of patients) that have a particular prognostic significance,^52^,^53^ diagnosis may be very difficult using bone marrow aspirates. In this regard, a scoring system for the differential diagnosis between MDS and other myeloid neoplasms with fibrosis, and between MDS and other cytopenias with reduced bone marrow cellularity was developed.^47^

Bone marrow biopsy also allows a better evaluation of megakaryocytes and may show the presence of aggregates or clusters of blasts, a typical finding in aggressive subtypes.^35^,^54^ Moreover, it can provide material for additional diagnostic procedures, such as immunohistochemistry, in situ hybridization or molecular analysis.

## Recommendations for Diagnosis

**Figure 3:**
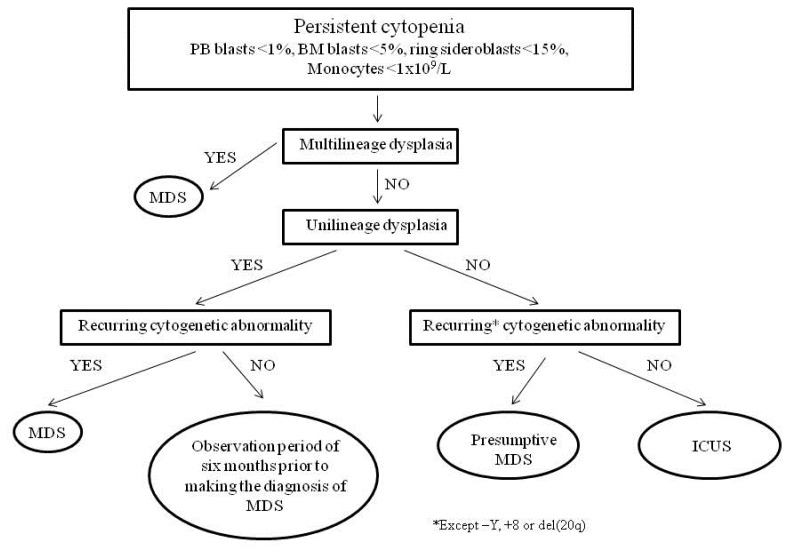
Diagnostic algorithm.

The combination of manifest bone marrow dysplasia and clonal cytogenetic abnormality allows a conclusive diagnosis, but this is possible for only a part of patients. Diagnosis may be particularly difficult in patients with <5% bone marrow blasts and only one cytopenia. If a patient with a clinical and laboratory suspect of MDS has inconclusive morphological features, a presumptive diagnosis of MDS can be made in the presence of a specific chromosomal abnormality demonstrating clonality. If there is only unilineage dysplasia, in the absence of recurrent cytogenetic abnormalities, without increase of peripheral or bone marrow blasts, with less than 15% ring sideroblasts, an observation period of 6 months and repeating bone marrow examination is recommended prior to making the diagnosis of MDS. For patients with persistent cytopenia(s) (at least 6 months), in the absence of morphological or cytogenetic evidence sufficient for a definitive diagnosis of MDS, the term “idiopathic cytopenia of undetermined significance” (ICUS) should be used (Figure 3).

## Newly Defined Entities

The term ICUS was first proposed by the IWGM-MDS at a meeting in Lisbon in 2005, and subsequently used in the 2008 WHO classification and by others. ICUS and idiopathic dysplasia of undetermined significance (IDUS) are conditions in which the criteria for the diagnosis of MDS are not satisfied, even if cytopenia or dysplasia is present.^6^,^55^–^58^ ICUS is characterized by persistent primary cytopenia, in the absence of morphological or cytogenetic abnormalities specific of MDS, whereas in IDUS there are morphological and/or karyotypic dysplastic alterations, casually observed, in the absence of cytopenia. In ICUS, cytopenia may concern one or more hematopoietic lineages; therefore, the terms of idiopathic anemia, neutropenia, thrombocytopenia, or bi/pancytopenia of uncertain significance have been proposed. The groups of cases so far described are numerically small, except the one obtained from the MDS registry of Düsseldorf.^59^ In both ICUS and IDUS, a neoplastic clone can be found already at diagnosis, and progression to an overt MDS or another myeloid malignancy is possible after a variable period. Thus, these conditions should be considered as a potential pre-phase of myeloid neoplasms, and have to be closely monitored for the unpredictable course.

## Conclusions

Despite the WHO diagnostic and classification criteria, the morphological diagnosis of MDS is still often critical and requires considerable expertise.^60^ On the other hand, as more specific treatments are becoming available, an accurate diagnosis is increasingly important. Recently, the use of new molecular techniques, including gene expression profiling and analysis of point mutations, has allowed to detect, even in patients with normal karyotype, clonal abnormalities of considerable diagnostic and prognostic meaning.^61^–^64^ However, although in the future morphology and cytogenetics should be integrated with the new molecular techniques to classify MDS,^65^ for the moment the morphological approach continues to be fundamental at least at the beginning of the diagnostic algorithm.

## Figures and Tables

**Table 1:** Differential diagnosis.

Therapy-related MDS (cytotoxic therapy, irradiation) Drug-induced cytopenias B12/folate deficiency, zinc/copper deficiency Excessive alcohol intake Exposure to heavy metals (lead, arsenic) Infections (HIV, Epstein-Barr virus, hepatitis C, parvovirus, leishmania) Hemophagocytic lymphohistiocytosis Anemia of chronic disorders (infection, inflammation, cancer) Autoimmune cytopenia Metabolic disorders (liver failure, kidney failure) Other hematopoietic stem cell disorders (acute myeloid leukemia, myeloproliferative neoplasms, aplastic anemia, paroxysmal nocturnal hemoglobinuria, LGL leukemia) Constitutional disorders (congenital dyserythropoietic anemia, sideroblastic anemia, Fanconi’s anemia, Down syndrome)

**Table 2:** Recurrent chromosomal abnormalities and their frequency in MDS.^6^

Unbalanced abnormality	Frequency (%)	Balanced abnormality	Frequency (%)

+8	10	t(11;16)(q23;p13.3)*	
-7 or del(7q)*	10	t(3;21)(q26.2;q22.1)*	
-5 or del(5q)*	10	t(1;3)(p36.3;q21.2)*	
del(20q)	5–8	t(2;11)(p21;q23)*	1
-Y	5	inv(3)(q21q26.2)*	1
i(17q) or t(17p)*	3–5	t(6;9)(p23;q34)*	1
-13 or del(13q)*	3		
del(11q)*	3		
del(12p) or t(12p)*	3		
del(9q)*	1–2		
idic(X)(q13)*	1–2		

* In the setting of persistent cytopenia of undetermined origin, these abnormalities are considered presumptive evidence of MDS.

**Table 3:** WHO-2008 classification of MDS.^6^

Name	Abbreviation	Peripheral blood	Bone marrow	Proportion of MDS patients

Refractory cytopenia with unilineage dysplasia	RCUD	<1% blasts	<5% blasts Dysplasia in ≥10% cells	
Refractory anemia	RA	Anemia	Unilineage erythroid dysplasia Unilineage granulocytic dysplasia	10%–20%
Refractory neutropenia	RN	Neutropenia	Unilineage megakaryocytic dysplasia	<1%
Refractory thrombocytopenia	RT	Thrombocytopenia		<1%
Refractory anemia with ring sideroblasts	RARS	Anemia No blasts	<5% blasts Unilineage erythroid dysplasia ≥15% of erythroid precursors are ring sideroblasts	3%–11%
Refractory cytopenias with multilineage dysplasia	RCMD	<1% blasts Cytopenia(s) No Auer rods	<5% blasts Multilineage dysplasia ± ring sideroblasts No Auer rods	30%
MDS, unclassifiable	MDS-U	Cytopenias ≤1% blasts	Dysplasia and <5% blasts If no dysplasia, MDS-associated karyotype	?
MDS-associated with isolated del(5q)	Del(5q)	Anemia Normal or high platelet count <1% blasts	Normal to increased megakaryocytes with hypolobated nuclei Isolated del(5q) <5% blasts No Auer rods	Uncommon
Refractory anemia with excess blasts, type 1	RAEB-1	Cytopenia(s) <5% blasts No Auer rods	Uni- or multilineage dysplasia 5–9% blasts No Auer rods	
Refractory anemia with excess blasts, type 2	RAEB-2	Cytopenia(s) 5–19% blasts ± Auer rods	Uni- or multilineage dysplasia 10–19% blasts ± Auer rods	40%

**Table 4:** Morphological features of myelodysplasia.^2^,^6^

Lineage dysplasia	Peripheral blood	Bone marrow

***Dyserythropoiesis***	Anisocytosis Poikilocytosis Basophilic stippling	*Nuclear* Nuclear budding Internuclear bridging Karyorrhexis Multinuclearity Nuclear hyperlobation Megaloblastic changes *Cytoplasmic* Ring sideroblasts Vacuolization Periodic acid-Schiff positivity Inclusions Incomplete hemoglobinization Fringed cytoplasm
***Dysgranulopoiesis***	Granulocyte nuclear hypolobation (pseudo Pelger-Hüet) Granulocyte cytoplasmic hypo/degranulation Blasts	Anisocytosis Nuclear hypolobation (pseudo Pelger-Hüet) Irregular hypersegmentation Bizarre nuclear shapes Decreased granules; agranularity Pseudo Chediak-Higashi granules Auer rods
***Dysmegakaryocytopoiesis***	Platelet anisocytosis Giant platelets	Micromegakaryocytes Nuclear hypolobation Small binucleated elements Dispersed nuclei Degranulation

**Table 5:** Morphological score.^47^

Morphological abnormalities	Cut off values	Cohen’s K coefficient (inter-operator variability)	Variable weighted score
Dyserythropoiesis*			
Megaloblastosis	>5	.83	2
Bi- or multinuclearity	>3	.87	1
	>5		2
Nuclear lobulation or irregular contours	>3	.84	1
Pyknosis	>5	.81	1
Cytoplasmic fraying	≥7	.82	1
Ring sideroblasts	>5	.95	2
	≥15		3
Ferritin sideroblasts	≥30	.92	1

Dysgranulopoiesis*			
Myeloblasts	≥3%	.92	1
	≥5%		3
Auer rod	≥1%	.90	3
Pseudo Pelger-Hűet anomaly	>3%	.87	1
	>5%		2
Abnormal nuclear shape	≥7%	.86	1
Neutrophil hypogranulation	>3%	.81	1
	>5%		2

Dysmegakaryopoiesis*			
Micromegakaryocytes	>5%	.88	3
Small binucleated megakaryocytes	>5%	.81	1
Megakayocytes with multiple separated nuclei	>5%	.84	2
Hypolobated/monolobar megakaryocytes	>5%	.86	2

* Erythroid, myeloid and megakaryocytic dysplasia was defined in the presence of a score value ≥3.

**Table 6:** Diagnostic value and inter-observer reproducibility of the morphological score in an independent cohort of patients (MDS and non-clonal cytopenias).^47^

	Sensitivity %		Specificity %		Concordance between panel 1 and 2 (K test)

	*Morphologist panel 1*	*Morphologist panel 2*	*Morphologist panel 1*	*Morphologist panel 2*	

***Dyserythropoiesis***	92	87	91	89	.83
***Dysgranulopoiesis***	89	90	98	87	.82
***Dysmegakaryocytopoiesis***	89	86	99	94	.86
